# 2D shear wave elastography (SWE) performance versus vibration-controlled transient elastography (VCTE/fibroscan) in the assessment of liver stiffness in chronic hepatitis

**DOI:** 10.1186/s13244-020-0839-y

**Published:** 2020-03-10

**Authors:** Ahmed M. Osman, Ahmed El Shimy, Mohamed M. Abd El Aziz

**Affiliations:** Faculty of Medicine, Ain Shams University, Cairo, Egypt

**Keywords:** Chronic liver disease, Liver stiffness, Shear wave elastography, Fibroscan, Transient elastography

## Abstract

**Background:**

The assessment of liver stiffness and the degree of fibrosis are important factors affecting the management strategy. Multiple non-invasive tools are now available to offer an adequate alternative to biopsy. In this study, we tried to compare the performance of 2D shear wave elastography (SWE) to the transient elastography/fibroscan as a non-invasive tool in the prediction of liver stiffness. This is a prospective study of 215 patients confirmed by serology to have positive virus C or B infection. 2D SWE was done followed by vibration-controlled transient elastography (VCTE) known as fibroscan at the same session. Biopsy results were collected.

**Results:**

The mean age was 51.07 years ± 6.07 SD. Five cases were excluded due to insufficient data. Fibroscan failed in 30 cases out of 210 cases (failure rate of 14.3%) compared with only 12 patients (6.7% failure rate) while using SWE. Only 180 patients completed the study to the result analysis. SWE results showed significant agreement to the fibroscan results with 86.7% agreement with a tendency for overestimation of the degree of fibrosis (11.7%). The efficacy of SWE was the highest during the assessment of patients with F0 (98.9%), F1 (97.8%), and F4 (93.3%) respectively and relatively low in F2 (92.8%) and F3 (90.6%).

**Conclusion:**

2D SWE is a relatively recent non-invasive tool in the assessment of liver fibrosis grading which can be used as an alternative to the fibroscan with almost similar diagnostic performance especially when fibroscan is not capable to obtain adequate results such as in obesity and ascites.

## Key points

- Chronic liver disease is one of the commonest chronic diseases worldwide.
- The degree of fibrosis is important to determine the treatment strategy.
- SWE and fibroscan are non-invasive tools for liver fibrosis grading.
- SWE offers almost similar diagnostic accuracy as fibroscan with overestimation tendency.

## Background

Chronic liver disease (CLD) is one of the most common chronic diseases worldwide with multiple etiological factors and high morbidity and mortality rates [1]. CLD caused by multiple factors including alcohol, viral hepatitis, drug induced, auto-immune diseases, and obesity with all these factors leads to liver fibrosis/cirrhosis which may end to liver cell failure and death [2]. Viral hepatitis is one of the commonest causes of CLD especially in Egypt with hepatitis C comes on top of the causes of CLD in Egypt [3].

The management and treatment strategy of CLD depends on the clinical status of the patient, laboratory liver profile, and the degree of liver fibrosis [4]. Liver biopsy was considered the gold standard for assessment of the degree of liver fibrosis, but it carries a lot of risks being painful, expensive, and risk of hemorrhage comes on top of these complications [5].

As a result of liver biopsy complications, the search for non-invasive tools for assessment and grading of liver fibrosis is rising with transient elastography (TE) or fibroscan becomes the commonest non-invasive tool used as an alternative for biopsy. It is a non-invasive, bedside, and rapid test [2, 6].

TE probe uses a mechanical vibration that creates shear wave within the liver parenchyma and also ready to read the velocity reflected from the liver surface which shows changes according to the degree of liver fibrosis, then giving a measurement reflecting the degree of liver stiffness which is displayed in kilopascal (kPa) [7, 8]. TE or fibroscan has some limitations when used in patients with ascites, morbid obesity, and massive pleural effusion [9].

2D shear wave elastography (SWE) is relatively recent tool in the era of liver fibrosis grading as a non-invasive tool which uses the usual 2D US probe, yet with production of a focused acoustic beam to generate shear wave within the liver with the degree of liver fibrosis reflected upon the speed of this wave within the liver parenchyma and affects the degree of reflected wave. The same probe can track the reflected wave then calculate the degree of liver fibrosis using the machine software which is presented with meter per second [10, 11].

In this study, we tried to assess the diagnostic performance of SWE as a non-invasive tool in the detection of the degree of liver stiffness and fibrosis compared to the TE/fibroscan in patients with known CLD.

## Methods

### Patients

This was a prospective study conducted on randomized selected 215 patients known to have chronic hepatitis infection either hepatitis C or hepatitis B and came for liver fibrosis grading as pre-therapeutic assessment or follow-up during the management course. Each patient did the usual B-mode ultrasound followed by 2D SWE then TE or fibroscan which was done in the same session. The study was conducted over the period from March 2019 to October 2019. Written informed consent was taken from all patients to use their results data according to the ethical committee regulations.

### Inclusion criteria

All patients with chronic hepatitis and proved by laboratory data to have positive viral C or B infection. No age or sex predilection. Available hepatic biopsy results with histopathological fibrosis scoring with a maximum of three months before the scan time.

### Exclusion criteria

We excluded patients with congestive heart disease, acute hepatitis, hepatic, or portal vein thrombosis or anomalies, and any patient with hepatic focal lesion or history of a hepatic interventional procedure as radiofrequency ablation (RF) or chemoembolization. Also, patients with unavailable hepatic biopsy results or biopsies more than 3 months before the scan time.

### Patient preparation

The patient underwent fasting for 4–6 h. An adequate full history was taken. A documented laboratory result for hepatitis markers was available. A previous biopsy result also needed.

### Technique

#### US machine

LOGIQ S8 XDclear 2.0 with vibration controlled TE probe and the 2D SWE software. General Electric (GE) company, USA.

#### The technique for SWE

All patients were placed supine or left semi-lateral decubitus with the 2D convex probe placed on the midclavicular line or anterior axillary line in the intercostal spaces until obtaining the most adequate window for the liver. The right lobe was the selected region of the liver with a distance of about 2 cm to the nearby capsule and devoid of a large blood vessel as we could. The patients were asked to hold breathing to minimize breathing motion artifacts. Then, SWE was initiated for about 5 s on the selected liver area through which about two to three frames of SWE were obtained. A region of interest (ROI) was applied inside each frame of SWE away from any visualized artifacts to obtain the best quantitative measurements. Twelve adequate measurements were needed. The *V* median/IQR ratio was considered to be < 25% to ensure adequate results and readings.

#### The technique for VCTE or fibroscan

All patients were placed on a supine position with elevated arms resting above the head level. B-mode was used to select the best intercostal space level at the midaxillary line (best at the level of the xiphoid process) with the best visualization field for the liver. We activated the fibroscan probe and applied it at the selected point perpendicular to the skin surface. An adequate amount of gel was considered, and appropriate compression pressure was applied known by the pressure indicator green light appeared on the screen. Ten measurements were taken to obtain the median number in kilopascal. IQR/median ratio was considered to be < 25% to ensure adequate results and readings.

### Image interpretation

The three authors are well trained on elastography techniques with at least 3 years of experience and all were blinded by the tissue biopsy result. Each patient did the SWE first with one of the authors followed by TE by another one who was blinded about the result of SWE. All results of each patient were collected. The measurements of SWE were expressed by meter per second and interpreted into liver fibrosis staging according to Metavir scoring (Table 1). The SWE speed converted to kilopascal using Young’s formula (kPa = 3 pv^2^) with *p* = tissue density which is always considered 1000 kg/m^3^ and *v* = speed of SWE [12].

**Table 1:** The interpretation of the SWE results by meter per second and the liver fibrosis staging using Metavir scoring

Liver fibrosis staging	Metavir score	m/s
Normal	F0	< 1.47 m/s
Normal–mild	F1	1.47–1.48 m/s
Mild–moderate	F2	1.48–1.64 m/s
Moderate–severe	F3	1.64–1.76 m/s
Cirrhosis	F4	> 1.76 m/s

**Fig. 1:**
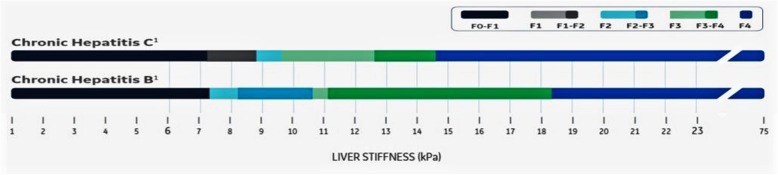
The interpretation of TE or fibroscan results by kilopascal and the liver fibrosis staging (quoted from GE healthcare documents, 2017)

Regarding the TE or fibroscan, the results were expressed by kilopascal which also interpreted into liver fibrosis staging according to Fig. 1 [13–16]. SWE results and the fibroscan results were compared to the liver tissue biopsy which was considered as the gold standard reference for liver fibrosis staging. Then, the SWE results were compared to the TE or fibroscan results while used as reference values.

### Analysis of data

The analysis of data was done using IBM SPSS statistics (v. 25.0, IBM Corp., USA, 2017-2018) was used for data analysis. Data were expressed as mean ± SD for quantitative parametric measures in addition to both number and percentage for categorized data.

The following tests were done:

1. Chi-square test to study the association between every 2 variables or comparison between 2 independent groups as regards the categorized data. The probability of error at 0.05 was considered sig., while at 0.01 and 0.001 are highly sig.
2. Diagnostic validity test includes sensitivity, specificity, and positive and negative predictive value as well as the efficacy.

## Results

This is a study conducted on 215 randomized selected patients with known CLD secondary to chronic hepatitis C or B infection. Only 180 patients completed the study to the analysis level. Five patients were excluded due to unavailable documented data about the hepatitis viral infection. Thirty patients representing 14.3% of cases were excluded due to failed fibroscan (19 patients due to obesity and 11 patients due to the presence of ascites). Eighteen of them showed successful SWE examination, while 12 patients also failed to do SWE (failure rate = 6.7%).

The mean age of the selected patients was 51.07 years ± 6.07 years SD. One hundred seventeen patients out of 180 were male representing 65% of the cases. Cases with positive virus C infection were 148 patients (82.2%), while cases with positive virus B infection were only 32 patients (17.8%). All the patients had available biopsy results with the median time between the biopsy and the scan time was 52 days.

Comparing the results of fibroscan and SWE with tissue biopsy showed an almost similar degree of agreement with no significant difference between the two techniques when compared with the biopsy results. However, SWE showed a higher incidence of overestimation (Tables 2 and 3). The agreement of fibroscan reached 90.6% compared with 87.2% of SWE. The degree of overestimation showed 5.6% in fibroscan while it was 10.6% in SWE. The degree of underestimation was 3.8% and 2.2% for fibroscan and SWE respectively. SWE showed a higher incidence of mismatch between patients with F4 (Table 3).

**Table 2:** The results of the fibroscan fibrosis score between the selected population compared to the tissue biopsy

			Fibroscan (VCTE) fibrosis score					Total
			F0	F1	F2	F3	F4	
Liver biopsy score	F0	Count	33	2	0	0	0	35 (19.4%)
	F1	Count	2	26	1	1	1	31 (17.2%)
	F2	Count	1	1	28	2	0	32 (17.8%)
	F3	Count	0	1	0	32	3	36 (20%)
	F4	Count	0	0	1	1	44	46 (25.6%)
Total		Count	36	30	30	36	48	180
		%	20%	16.7%	16.7%	20%	26.7%	100.0%

**Table 3:** The results of the SWE fibrosis score between the selected population compared to the tissue biopsy

			2D SWE fibrosis score					Total
			F0	F1	F2	F3	F4	
Liver biopsy score	F0	Count	32	2	1	0	0	35 (19.4%)
	F1	Count	1	24	1	3	2	31 (17.2%)
	F2	Count	1	1	27	0	3	32 (17.8%)
	F3	Count	0	1	0	28	7	36 (20%)
	F4	Count	0	0	0	0	46	46 (25.6%)
Total		Count	34	28	29	31	58	180
		%	18.9%	15.6%	16.1%	17.2%	32.2%	100.0%

The diagnostic accuracy of both techniques showed no significant difference when compared to the liver biopsy results with almost similar and close efficacy results as seen in Table 4.

**Table 4:** The diagnostic validity of fibroscan (TE) and SWE compared to tissue biopsy at different fibrosis scores

		F0			F1		F2		F3		F4	
		TE		SWE	TE	SWE	TE	SWE	TE	SWE	TE	SWE
	Sensitivity (%)		94.3	91.4	83.9	77.4	87.5	84.3	88.9	77.8	95.7	100
	Specificity (%)		97.9	98.6	97.3	97.3	98.6	98.6	97.2	97.9	97	91
	NPV (%)		98.6	97.9	96.7	95.4	97.3	96.7	97.2	94.6	98.5	100
	PPV (%)		91.6	94.1	86.7	85.7	93.3	93.1	88.9	90.3	91.7	97.3
	Efficacy (%)		97.2	97.2	95	93.9	96.7	96.1	95.6	93.9	96.7	93.3

Regarding fibroscan (VCTE) fibrosis score, patients with F4 were the comments representing 48 patients (26.7%) followed by F3 and F0 with both of them found in 36 patients (20%). F2 and F1 were the least with each found between 30 patients (16.7%) (Table 5).

**Table 5:** The results of fibrosis score between the selected population using both fibroscan and SWE technique with the fibroscan results used as the reference

			Fibroscan (VCTE) fibrosis score					Total
			F0	F1	F2	F3	F4	
SWE fibrosis score	F0	Count	34	0	0	0	0	34 (18.9%)
	F1	Count	1	27	0	0	0	28 (15.6%)
	F2	Count	1	3	23	2	0	29 (16.1%)
	F3	Count	0	0	5	25	1	31 (17.2%)
	F4	Count	0	0	2	9	47	58 (32.2%)
Total		Count	36	30	30	36	48	180
		%	20%	16.7%	16.7%	20%	26.7%	100.0%
Chi-square tests								
			Value	*P*				
Pearson chi-square			514.551	0.000				

When comparing the results of SWE to the fibroscan results, we found 86.7% agreement with 11.7% overestimation and 1.7% underestimation. *P* value was < 0.001 denoting a highly significant correlation between the results of SWE and the results of fibroscan.

The highest incidence of mismatch was found between patients with F3 fibroscan score (11 cases) followed by patients with F2 fibroscan score (7 cases), while the mismatch was the least between patients with fibroscan score F0 and F1 (2 and one respectively) (Table 5).

**Fig. 2:**
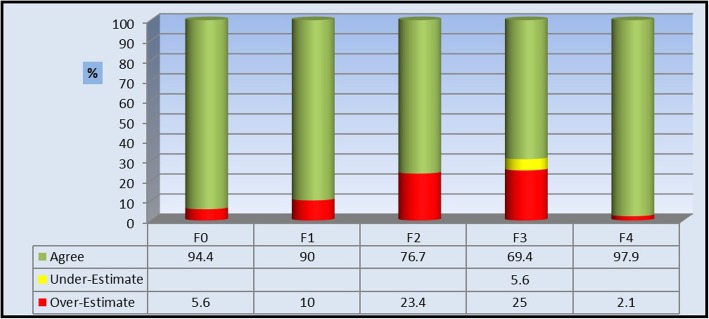
The degree of agreement and the mismatch incidence between the SWE fibrosis score compared to the fibroscan fibrosis score

**Fig. 3:**
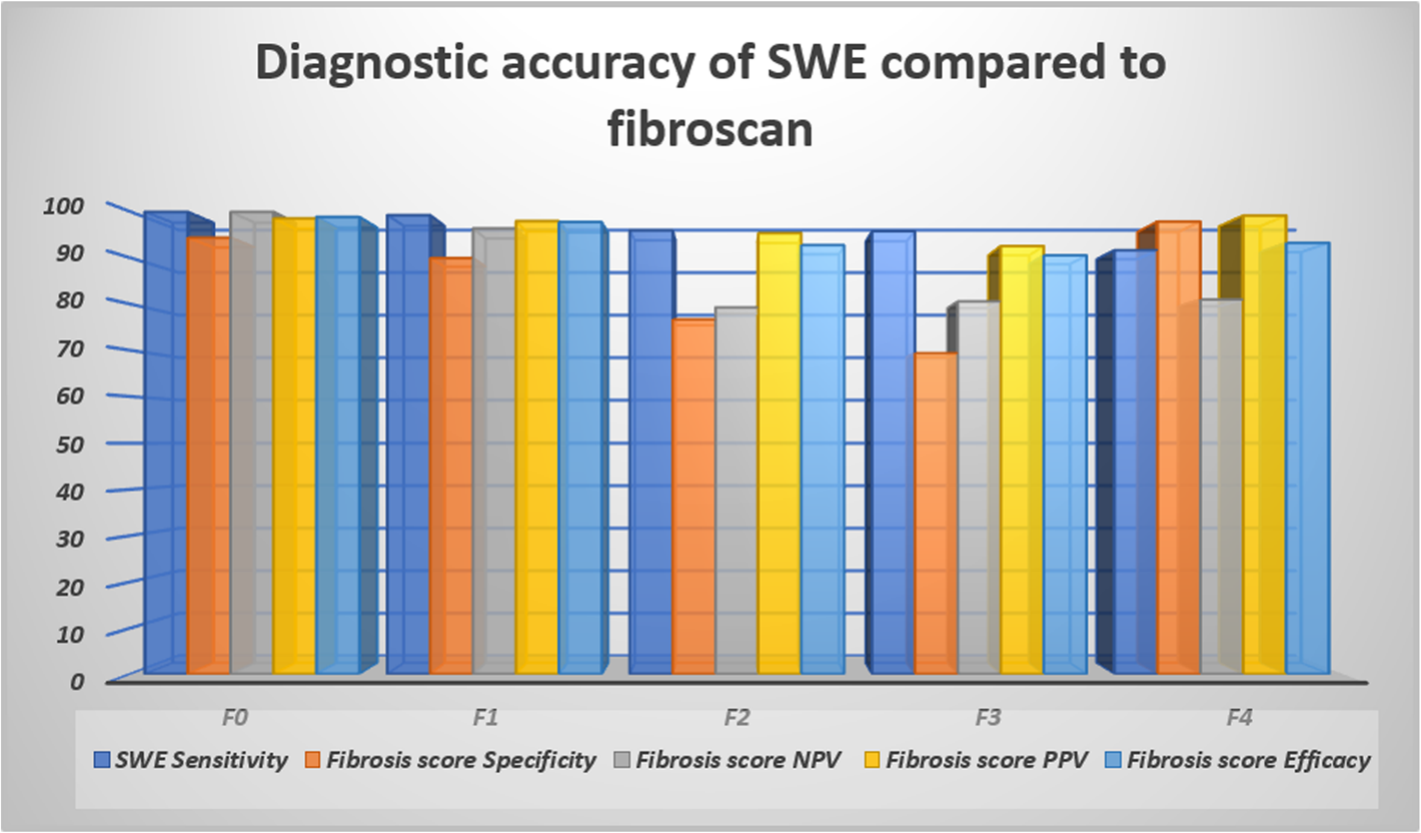
The diagnostic accuracy of SWE compared to the TE fibroscan when using the TE fibroscan results as a reference

**Fig. 4:**
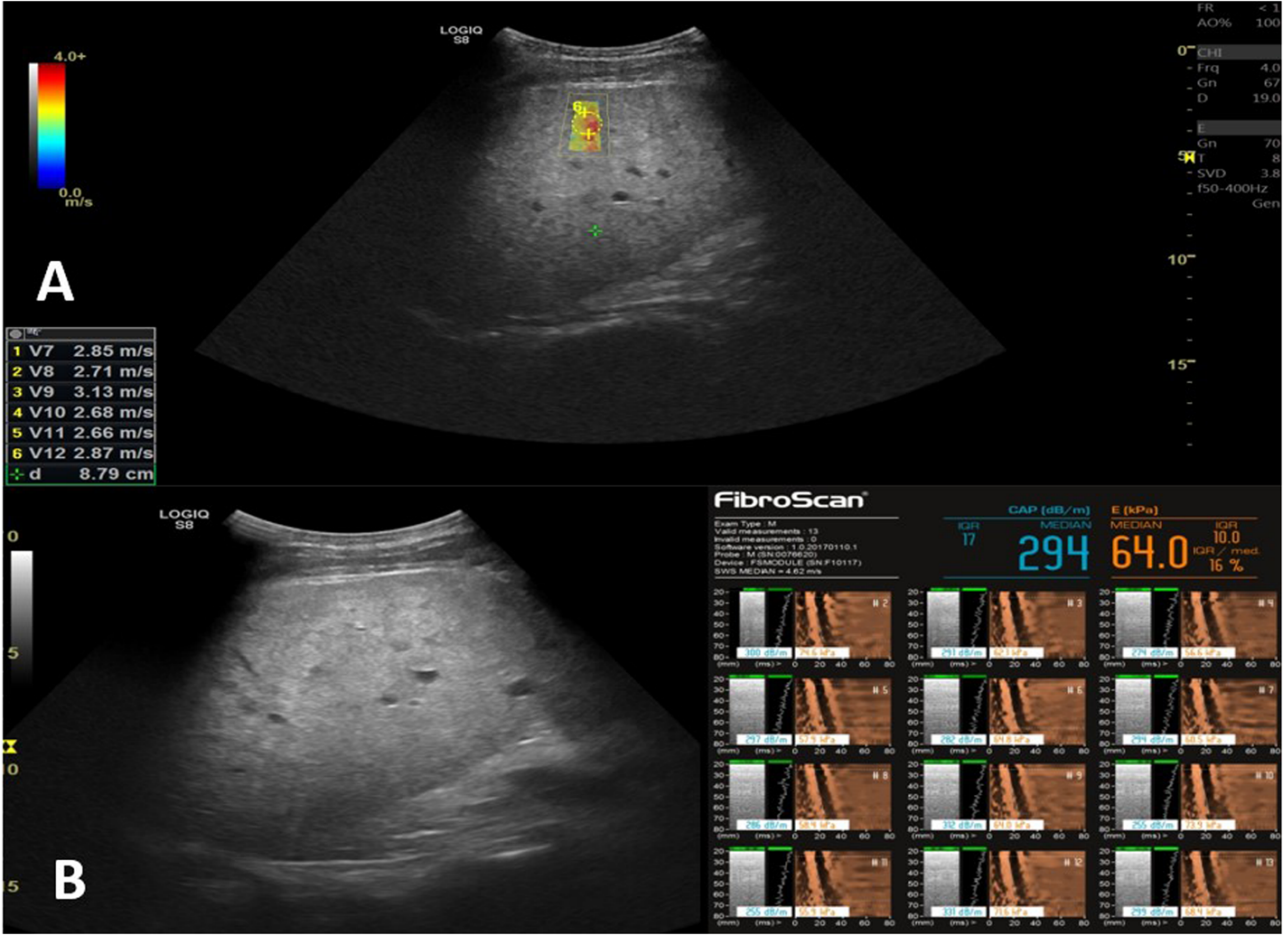
A male patient 52 years old with chronic hepatitis C infection on follow-up. **a** SWE revealed median velocity = 2.62 m/s and *V* median/IQR = 14.6% consistent with F4 according to Metavir score. **b** Fibroscan was done for the same patient and revealed kPa = 64 and IQR/median = 16% consistent with F4

**Fig. 5:**
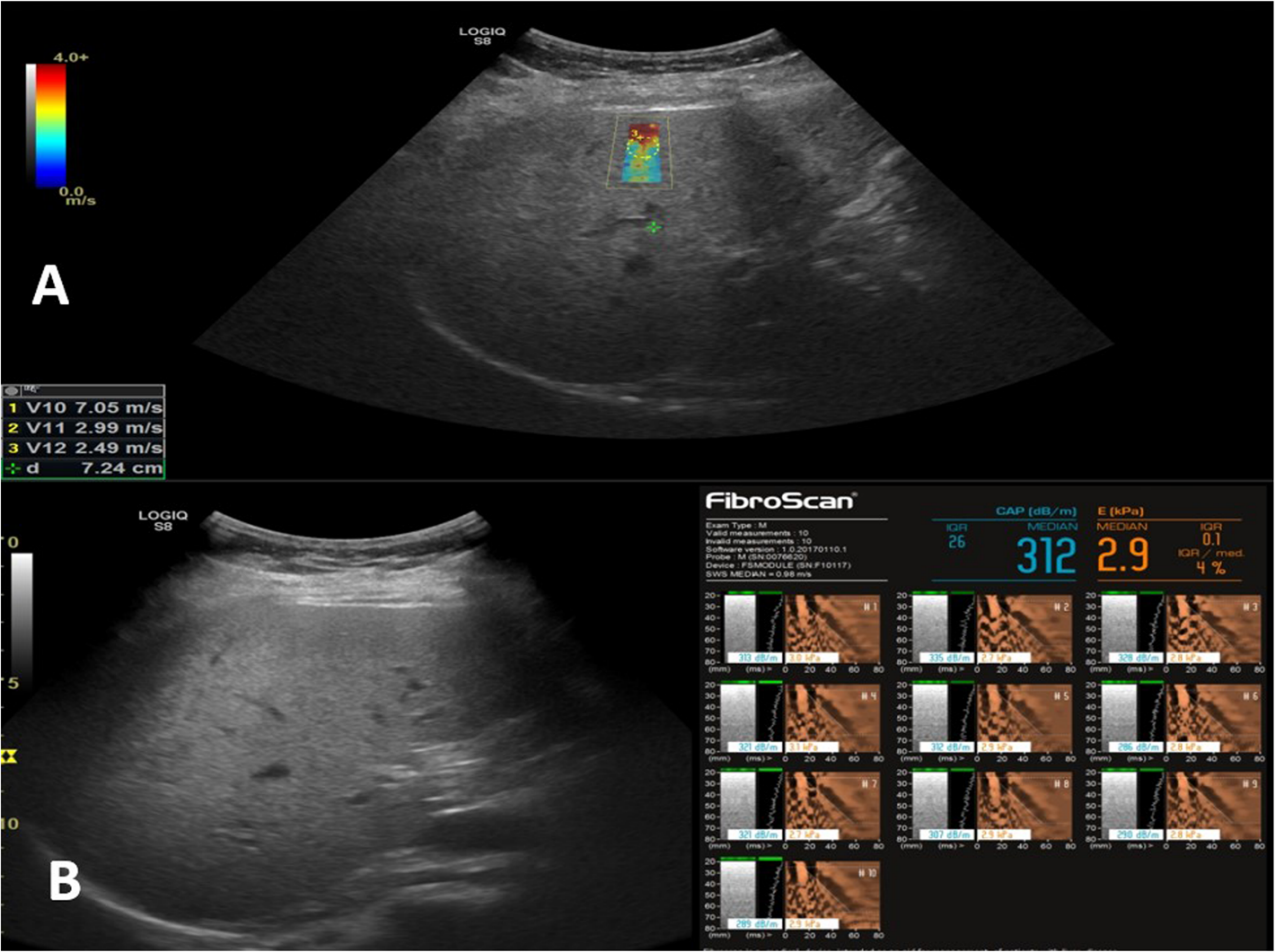
A female patient 48 years old with chronic hepatitis B infection on follow-up. **a** SWE revealed median velocity = 1.54 m/s and *V* median/IQR = 23% consistent with F2 according to Metavir score. **b** Fibroscan was done for the same patient and revealed kPa = 2.9 and IQR/median = 4% consistent with F0

The SWE tends to overestimate the results fibrosis score when compared to the fibroscan with the highest degree of overestimation found at F3 and F2 patients. SWE shows high efficacy in all degrees of fibrosis with the lowest found at F3 and F2, while the highest efficacy found at F0 and F1 (Figs. 2, 3, 4 and 5) (Table 6).

**Table 6:** The diagnostic validity of SWE compared to the fibroscan at different fibrosis scores

			F0	F1	F2	F3	F4
SWE fibrosis score	Sensitivity		100	99.3	96	95.8	91.7
	Specificity		94.4	90	76.7	69.4	97.9
	NPV		100	96.4	79.3	80.6	81
	PPV		98.6	98	95.4	92.6	99.2
	Efficacy		98.9	97.8	92.8	90.6	93.3
Pearson chi-square		Value	167.671	151.883	97.674	86.084	129.346
		*P*	0.000	0.000	0.000	0.000	0.000

The duration of the fibroscan was 5.3 ± 2.2 min, while it was 5.4 ± 2.5 min for SWE with no significant difference between the two techniques regarding the duration of the technique.

## Discussion

CLD secondary to chronic hepatitis infection is one of the most common chronic debilitating diseases in Egypt, and the assessment of the degree of liver fibrosis is important in patient management. Recently, the liver biopsy no longer becomes the main tool for liver fibrosis staging to avoid its complications and hazard especially with the advanced progress in the non-invasive ultrasound tools. Different US elastography techniques now became essential in the management of patients with CLD and offer an adequate alternative to biopsy according to the World Federation for the US in medicine and biology guidelines [17].

TE or fibroscan is considered one of the most used alternatives for biopsy and already put in patient’s management algorithms in most of the European countries. However, some limitation was observed with the use of TE or fibroscan regarding obesity and ascites [7, 18].

In our study, the failure rate of fibroscan reached about 14.3% secondary to two reasons namely the ascites and patient’s obesity. Out of fibroscan failed cases, 18 cases were examined successfully using SWE with adequate results confirmed with IQR/median ratio < 25%, while the rest 12 also failed by SWE with a failure rate of SWE = 6.7%. This is already consistent with similar studies as Castera et al [19] who reported a failure rate of fibroscan reaching 20% with overall 5 years experience with patient’s obesity was considered one of the most important causes of technique failure. Foucher et al [20] recorded a less failure rate of fibroscan 6.2% which is much lower than our result, but they found also that high body mass index was the commonest failure cause. Zeng et al [21] recorded less failure rate of SWE compared with fibroscan but with lesser incidence compared with our study being 7% regarding the fibroscan and 1.9% regarding the SWE. The difference in failure rate taking into consideration the obesity being the commonest cause may vary between the different studies depending on the race and their body mass configuration.

The lower failure rate of SWE can be explained by the simultaneous B-mode visualization available during the SWE technique which provides the opportunity to select the proper site for reading away from the ascites interface and can avoid areas of obesity which was more common in the axillary region rather than the anterior chest wall in our patients. This is in controversy to the fibroscan which depends on the application of the probe blindly on a specific anatomical level on the patient’s body at the midaxillary level which showed more fat level rather than the anterior chest wall.

Our methodology was almost similar to O’Hara et al [1], Ryu et al [22], and Roccarina et al [23] who also compared the performance of SWE compared to TE with TE results was the reference values. However, their study was on a fewer sample volume compared with our study which included 180 patients. O’Hara et al [1] results were similar to our study and they concluded that SWE showed almost similar accuracy as TE, but SWE showed a tendency to overestimate the TE results.

Multiple studies [4, 10, 24, 25] tried to assess the accuracy of SWE compared with TE/fibroscan, yet they used the liver biopsy as a reference which was also applied in our study.

SWE is a recent modality used for fibrosis assessment which allows real B-mode visualization of the selected area with a larger area of selection under analysis compared with fibroscan. In this study, we assessed the diagnostic performance of 2D SWE compared with the usual TE or fibroscan in the assessment of liver fibrosis in patients with CLD secondary to chronic hepatitis infection. We found high significant agreement between the results of SWE and the results of fibroscan with high diagnostic performance and efficacy results between different F score results obtained by fibroscan with 11.7% overestimation tendency noted mainly between the patients with F3 and F2 (Tables 5 and 6 and Fig. 2).

Ali Z and colleagues [4] concluded a strong correlation and agreement between SWE and TE results with Kendalls *t-b* was 0.902, Spearman’s *p* value was 0.947, and the weighted *k* test value was 0.873.

Leung et al [24] compared SWE results to liver biopsy with 85% and 92% SWE sensitivity and specificity respectively in the diagnosis of liver fibrosis as well as 97% and 93% sensitivity and specificity in the diagnosis of liver cirrhosis, and this was close to our results as seen in Table 4.

Tada et al [10] and Ferraioli et al [25] stated that SWE can be used similar to TE in the assessment of liver fibrosis. Deffieux et al [26] found that the results of SWE are almost equal or even better than the results of TE.

Zeng et al [21] compared the SWE and TE to liver biopsy in patients with chronic hepatitis B infection and found SWE had a higher rate of reliability 98.1% than TE (93%). They found also a strong correlation between SWE and TE with no difference between the area under ROC curves of SWE and TE for liver fibrosis staging. Zhuang et al [27] showed that the diagnostic performance of SWE, namely the sensitivity and specificity, were higher in the diagnosis of F4 more than F2 and F3 which is almost similar to our results as noted in Table 4; SWE showed higher sensitivity, negative predictive value (NPV), and positive predictive value (PPV) in patients with F4 compared to the other patients when compared to tissue biopsy.

European Federation for societies for ultrasound in Medicine and Biology recommended the SWE to assess the degree of liver stiffness in patients with CLD secondary to hepatitis especially hepatitis C [28].

The limitation of our study was the absent control group due to the difficulty to get a biopsy from a healthy person. Also, still, the US is operator dependent, and we didn’t assess the inter-operator variability which of course further studies are needed to study this issue.

## Conclusion

SWE shows almost similar diagnostic performance compared to fibroscan which considered recently as a main non-invasive tool in liver fibrosis staging with minimal tendency to overestimate the degree of fibrosis. SWE can be used with high performance as an alternative to fibroscan especially when fibroscan is not able to obtain adequate results as in obesity and massive free ascites. Also, SWE gives the operator a real-time visualization of the selected area with a large surface area compared to fibroscan.

## Data Availability

Available on request with the corresponding author.

## Abbreviations

CLD: Chronic liver disease
NPV: Negative predictive value
PPV: Positive predictive value
SWE: Shear wave elastography
TE: Transient elastography
US: Ultrasound
